# Hydrogen cyanide acts as a regulator of reactive oxygen species metabolism

**DOI:** 10.1007/s00425-025-04848-1

**Published:** 2025-10-28

**Authors:** Maciej Piekarniak, Leslie A. Weston, Agnieszka Gniazdowska, Urszula Krasuska

**Affiliations:** 1 Department of Plant Physiology, Institute of Biology, Warsaw University of Life Sciences–SGGW, Nowoursynowska 159, 02-776 Warsaw, Poland; 2 Gulbali Institute for Agriculture, Water and the Environment, Charles Sturt University, Wagga Wagga, NSW 2678 Australia

**Keywords:** Gasotransmitters, Reactive oxygen species, Oxidative stress, Antioxidants, Free radicals

## Abstract

**Main conclusion:**

Hydrogen cyanide (HCN) is a ubiquitous gasotransmitter essential for regulating ROS metabolism and cellular redox balance. This modulation plays a crucial role in metabolic processes in higher plants and animals, highlighting HCN’s importance in cellular signalling and stress response.

**Abstract:**

Hydrogen cyanide (HCN) is synthesised in plants and animals and present ubiquitously in the environment. It is considered to be a gasotransmitter and is proposed to play a fundamental role in the origin of life. At concentrations higher than 100 µM, HCN is highly toxic to most aerobes, but at lower concentrations (below 100 µM) it serves as a signalling molecule in plants. The importance of this molecule in plant metabolism is highlighted by the fact that all higher plants produce HCN via various pathways. Given its toxicity, plants frequently store HCN as conjugates with sugars or lipids in vacuoles. HCN modulates the metabolism of reactive oxygen species (ROS), and this is also linked to the disruption of electron flow in the mitochondrial respiration chain. ROS are signalling compounds acting together with hormones in regulation of many physiological processes and typically modify the activity of enzymatic antioxidants by altering ROS levels, thereby impacting cellular redox potential. The aim of this review, therefore, is to describe the relationship between HCN activity and ROS metabolism, with a focus on higher plant systems in particular.

## Introduction

Cyanides are compounds with one carbon atom triple-bonded to a nitrogen atom (C≡N). Hydrogen cyanide (HCN) is a highly reactive molecule and a potent inhibitor of respiration or poison. This invisible colourless gas may be noticeable due to its scent, similar to that of bitter almonds (Raza and Jaiswal 1994). The physicochemical properties include its exceptional solubility in water (at 20 °C), which leads to a decrease in pH, with a pKa of 9.31 (Newhouse and Chiu 2010; Das et al. 2019). HCN is also soluble in alcohol, ether, glycerol, chloroform, and benzene. Its vapour pressure is comparable to that of air (0.948), and at high concentrations is flammable and explosive (Raza and Jaiswal 1994; Dagaut et al. 2008).

**Fig. 1:**
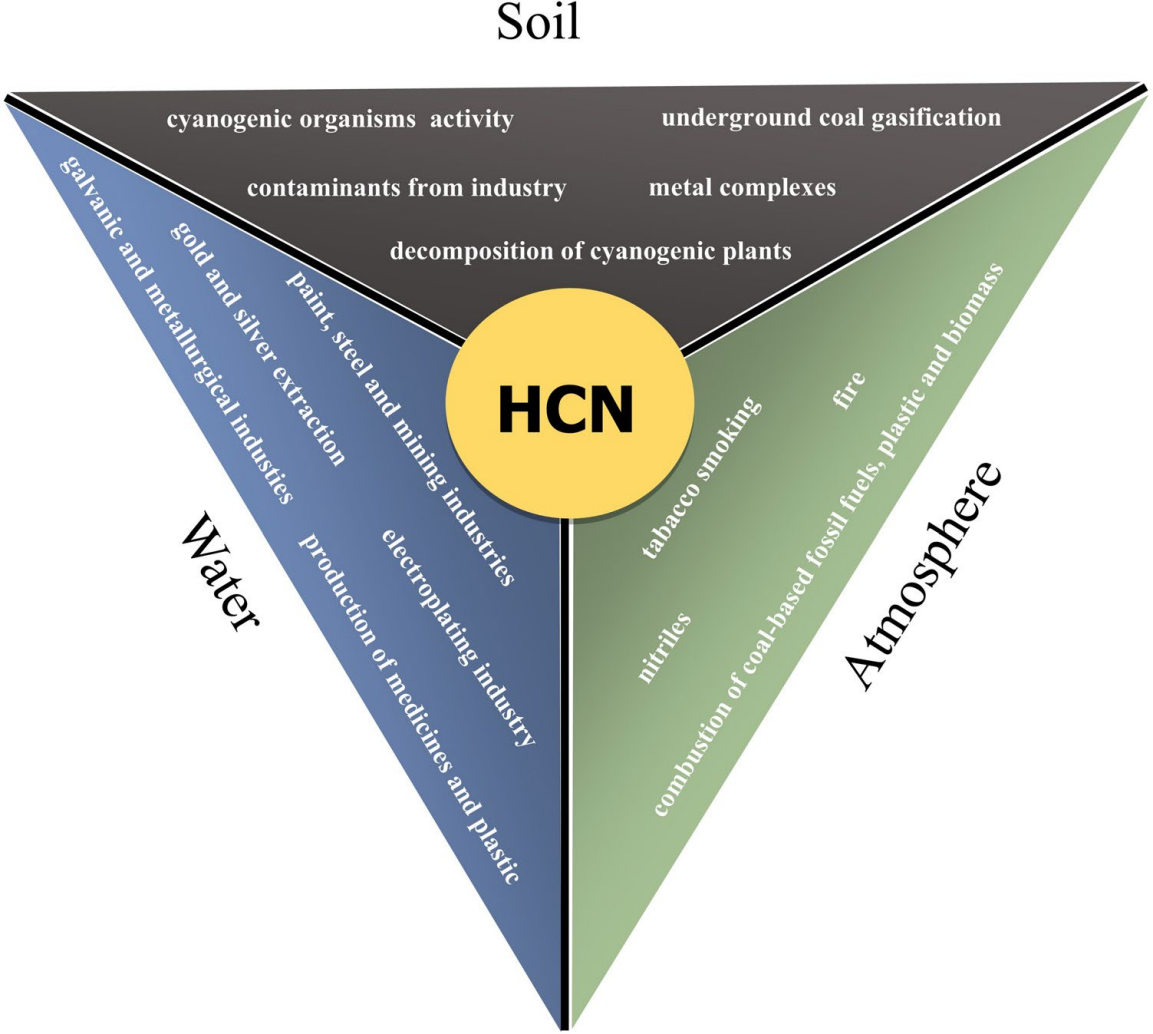
The main sources of HCN in the environment: water, soil and atmosphere

HCN has been implicated in the origin of life on Earth (prebiotic chemistry) (Das et al. 2019). A recent computational study, with the aid of the ab initio nanoreactor (AINR), suggested HCN and water as the first “Adam and Eve” molecules based on their potential for chemical evolution. These molecules are believed to have been the first chemical entities that could have possibly formed the precursors of RNA and proteins: cyanamide, glycolaldehyde, an oxazole derivative, and glycine (the proteinogenic amino acid) (Das et al. 2019). The presence of various forms of cyanide in the atmosphere, in living organisms, and in the soil has been well documented (Gleadow and Møller 2014; Wei et al. 2020; Cowan et al. 2021; Bruno et al. 2023) (Fig. 1). The properties of HCN, its production and biosynthetic pathways in animals and plants are also the topics of several more recent reviews (Siegień and Bogatek 2006; Jaszczak et al. 2017; Zuhra and Szabo 2022; Díaz-Rueda et al. 2023; Krasuska et al. 2023; Martinez and Diaz 2024).

HCN is produced by approximately 3000 different plant species from over 130 plant families (Yeats 2018). It is typically released from plant tissues through the enzymatic activity of *β*-glucosidase with cyanogenic glycosides (Emad Fadoul et al. 2023). Its toxicity is mainly due to the formation of stable complexes with transition metals of the prosthetic groups of metalloproteins essential for functionality (Nagahara et al. 1999). When ingested, HCN inhibits cytochrome *c* oxidase (COX) in the electron transport chain of mitochondria and thus it negatively affects the pathway responsible for production of ATP (Yeats 2018).

In plants, the activity of HCN has mainly been studied with respect to response to biotic stressors (Ebbs et al. 2010; Gleadow and Møller 2014; Cowan et al. 2021, 2022; Boter and Diaz 2023) and as a regulator of seed dormancy and germination (Roberts and Smith 1977; Esashi et al. 1981, 1991, 1996; Cohn and Hughes 1986; Oracz et al. 2008; Krasuska and Gniazdowska 2012). HCN has also been implicated in nitrogen metabolism by competitive inhibition of nitrogenase and inactivation of nitrate reductase (Ebbs et al. 2010; Blomstedt et al. 2018; Myrans et al. 2021). HCN, although commonly known as a toxic compound, is increasingly recognised as a signalling molecule in plants, playing a role in regulating various physiological processes like plant development, defence against pathogens, and response to environmental stresses. This molecule is also capable of reacting with cystine to form an organic thiocyanate (Gawron 1966). In proteins, HCN modifies cysteine residues by *S*-cyanylation, which serves as a new post-translational modification naturally occurring in plants (García et al. 2019).

At high levels, HCN remains highly toxic to plant cells (García et al. 2019; Díaz-Rueda et al. 2023). Depending on its concentration, it can act either as a toxin or a signalling molecule and is provided to plants either exogenously (in vitro or by cyanogenic bacteria present in the rhizosphere) or endogenously (in multiple reactions involving ethylene, camalexin, or other cyanide-containing compounds) (Díaz-Rueda et al. 2023).

Although COX is the main target of HCN, where electron transfer is blocked (Donato et al. 2007), HCN also affects chloroplastidic photosynthetic enzymes (Berg and Krogmann 1975). Both ribulose-bisphosphate carboxylase and plastocyanin are sensitive to HCN (Siegień and Bogatek 2006). HCN impacts other cellular enzymes, including catalases and oxidases (McMahon et al. 1995; Cheeke 1995). The response can be very rapid; for example, 20 min exposure for 50 µM HCN in 2-week-old *Arabidopsis thaliana* Heynh seedlings induced a stress response, manifested as a decrease in growth rate and chlorophyll concentration (Smith and Arteca 2000).

More recently, the physiological activity of HCN has been investigated as a signalling molecule (gasotransmitter) at concentrations below 100 µM (Zuhra and Szabo 2022; Díaz-Rueda et al. 2023; Krasuska et al. 2023; Zuhra et al. 2025). Chemically, it is able to penetrate membranes easily due to its lipid solubility and small molecular size. It is enzymatically generated and, due to its toxicity, detoxifying activities are required to maintain concentrations at non-toxic levels. Other cellular messengers, such as reactive oxygen species (ROS) and reactive sulphur and nitrogen species, also act in a similar manner (Krasuska et al. 2023; Zuhra et al. 2025). Subsequent binding of cyanide ion (CN^−^) to COX results in the depletion of cellular ATP production, reduced energy charge, increased calcium ion accumulation, and loss of cellular homeostasis (Isom and Borowitz 2016). On the other hand, it has been observed recently that trace levels of HCN induce cellular proliferation in mammalian cell cultures via COX stimulation, increased respiration and ATP production (Randi et al. 2021; Pacher 2021).

HCN can readily interact with products of incomplete oxygen reduction. Excited oxygen derivatives are commonly found in all higher organisms. Despite numerous studies focused on HCN generation in plants and microbes, the relationship between HCN and the formation of ROS has not been fully described. Interestingly, the generation of HCN in higher organisms, including mammalian species, leads to the eventual formation of ROS. The presence of reactive oxygen derivatives results in an alteration of redox potential, and this then determines HCN efficacy in inhibiting COX (Solomonson 1981) and other enzymes. COX, in its reduced form, is more resistant to HCN than in its oxidised form. Moreover, in the presence of reductants, the decomposition of the cyanide-COX complex is also observed (Solomonson 1981). Isolated COX can oxidise CN^−^ to cyanyl radical (^⋅^CN) that was detected using the electron spin resonance spin-trapping technique. The enzymatic conversion of CN^−^ to the ^⋅^CN by COX was time-dependent and not affected by azide (N_3_^−^) (Chen et al. 1999). Peroxidase or chloroperoxidase can also use potassium cyanide (KCN) or sodium cyanide (NaCN) as a substrate, and ^⋅^CN can be readily formed in a single-electron oxidation in the presence of hydrogen peroxide (H_2_O_2_) (Moreno et al. 1988). The generation of such free radicals can also trigger significant oxidative stress (Chen and Schopfer 1999).

Therefore, the aim of this review is to discuss the effects and interactions among HCN and ROS in both plant systems and, to a lesser extent, animal systems. As ROS are highly reactive, bimodal and implicated more broadly in the generation of oxidative stress, an important global phenomenon, this review is not only timely but useful. The key question we wish to address is how the endogenous formation of HCN or exogenous HCN of e.g. anthropogenic sources impacts the generation of oxidative stress in living organisms? Are there any rules governing HCN–ROS cross-talk?

## Hydrogen cyanide in the environment

As all living organisms are impacted by their local environment, both biotic and abiotic factors influence their metabolism. HCN is used and generated in many manufacturing processes, including the mining industry. It has been estimated by the Cognitive Market Research that the global HCN market will expand at a compound annual growth rate (CAGR) of 2.90% until 2031 (Phagare 2024). The increased demand for HCN will undoubtedly lead to higher emissions of this gas into the environment.

The combustion process, especially of nitrogen-containing compounds, is an important source of cyanides in the atmosphere (Dagaut et al. 2008; Moussa et al. 2016) (Fig. 1). The highest risk of HCN exposure is due to anthropogenic sources, encountered during combustion of such compounds as nylon, rayon, polyvinyl chloride, polyurethane foam, polyester wadding, neoprene foam, rubber, plastics, and styrofoam. The industrial origin of HCN is associated with the nitriles (acetonitrile/methyl cyanide, CH_3_CN) - the compounds containing a cyanide group. The nitriles are frequently employed in the laboratory during chemical and biotechnology processing and polystyrene manufacturing. After entering the body, nitriles are metabolised by the liver, leading to the release of cyanide. Therefore, cyanide is the byproduct of nitrile metabolism, so rather than the nitrile itself, it is that causes toxicity (Schnepp 2006).

Coal-based fossil fuels, plastics, and biomass combustion accelerates HCN emissions in the troposphere (Schnepp 2006; Dagaut et al. 2008). In the presence of water, glyceronitriles realised from burned plant material are hydrolysed to cyanide (Flematti et al. 2011). It has been demonstrated that the victims of smoke inhalation during house fires can suffer from cyanide poisoning (Schnepp 2006). A recent study documenting the effect of the Grenfell Tower fire in central London in 2017 suggests that the possible cause of death of 72 victims was HCN poisoning. Its presence was observed in both soil and debris around the fire site (Stec et al. 2019). In 2016, the Grenfell Tower rainscreen façade system was installed, consisting of combustible polyisocyanurate (PIR) foam, and as it later turned out, PIR foam was the main source of HCN in the Grenfell Tower fire (McKenna et al. 2019). Smoking (mainly tobacco smoking) is also an additional anthropogenic source of cyanides in the atmosphere (Fig. 1). Smokers and passive smokers or bystanders are exposed to toxins, including HCN, during the smoking process. As demonstrated, the gas phase of tobacco smoke has 400–500 chemical constituents and some 3500 compounds (Hoffmann and Hoffmann 1997; Hecht 1999). HCN is estimated to constitute approximately 22% of the 500 mg of smoke inhaled from a single cigarette by the smoker (Jaszczak et al. 2017). HCN is also a precursor of dinitrogen oxide (N_2_O), which has a very long atmospheric life (120 ± 30 years) and is a very significant greenhouse gas (Dagaut et al. 2008).

Anthropogenic pollution of water and soil caused by CN^−^ exposure or formation is also associated with the galvanic and metallurgical industries, as well as underground coal gasification (Jaszczak et al. 2017) (Fig. 1). Cyanide-containing aqueous waste streams also originate from the electroplating industry, gold and silver extraction, and the production of medicines and plastic (Teixeira et al. 2013; Jaszczak et al. 2017). Furthermore, cyanide-polluted wastewater is generated by the paint, steel, and mining industries, which are excreted or released and are recognised as biocides having a very negative impact on aquatic organisms (Teixeira et al. 2013). Some of the technology used to remove cyanides from wastewater is based on oxidation reactions, but occurs under special non-physiological conditions, suggesting this process requires careful regulation. 99.8% reduction of CN^−^ was observed when 100 mg L^−1^ of initial CN^−^ was used in the presence of H_2_O_2_ and sodium hypochlorite (NaClO) in the molar ratio of 2:1 and pH 9 (25 °C) (Teixeira et al. 2013).

**Fig. 2:**
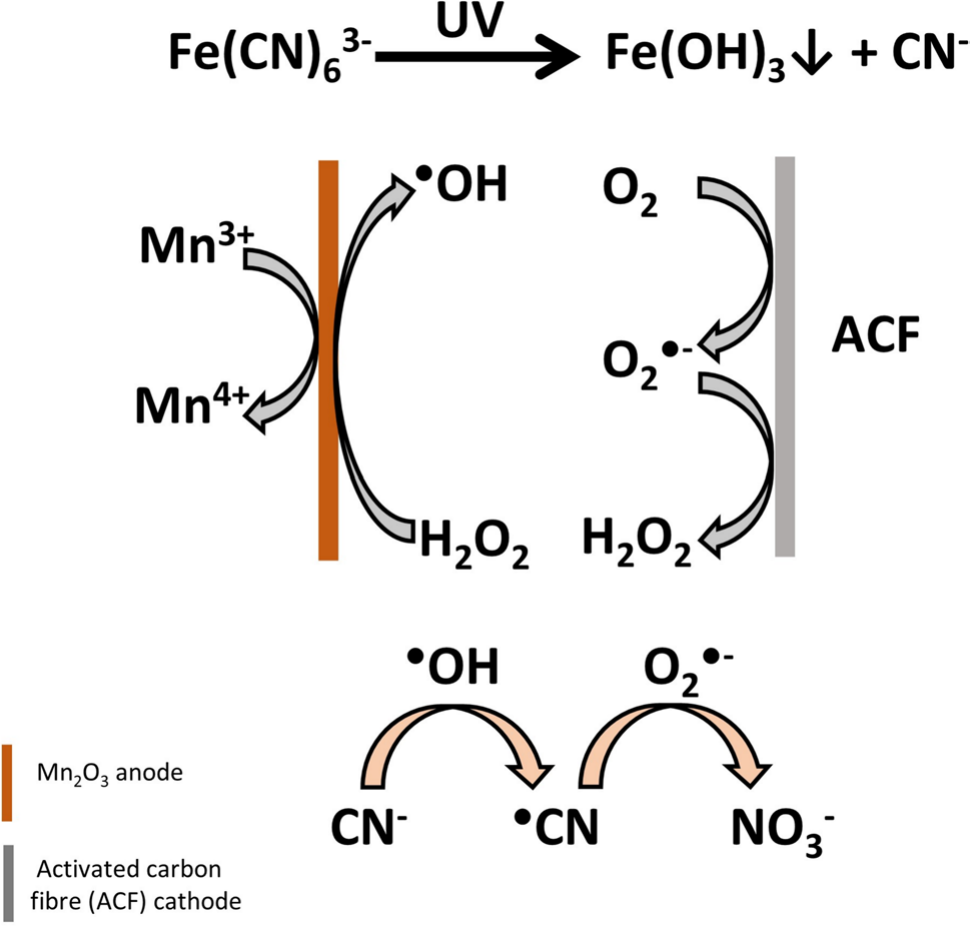
In the Electro–Fenton system, the formation of ^⋅^OH and O_2_^⋅−^ was observed by  using Mn_2_O_3_ anode and activated carbon fibre (ACF) cathode in the reactor connected to the potentiostat. The cyanide utilisation starts with the reaction of cyanide ions (CN^−^)present in the solution with ^⋅^OH, which generates cyanyl radical (^⋅^CN). ^⋅^CN may then be oxidised to nitrite ions (NO_3_^−^)^⋅^, providing cyanide removal, e.g. from wastewaters (based on Tian et al. 2022)

In the environment, HCN, as noted previously, can form a variety of metallo-cyanide complexes; the most common and stable being the iron cyanides, often found as soil contaminants on industrial sites and as a common chemical form of iron in groundwater (> 1% DW) (Meeussen et al. 1992; Samiotakis and Ebbs 2004) (Fig. 1). Investigators have shown that CN^−^ also forms complexes with metals such as iridium, platinum, palladium, zinc, germanium, mercury or cobalt (Castilla-Acevedo et al. 2020; Chen et al. 2020; Andrews and Cho 2021; Al-Jibori et al. 2022). The reaction kinetics of iron ion (Fe^3+^) forming complexes with KCN were also recently described by Kurashova and Kamyshny (2019). NaCN and copper ion (Cu^2+^) binding mechanisms were also investigated in association with UV/persulfate and UV/H_2_O_2_ (Sarla et al. 2004). The toxicity of such metallo-cyanide complexes is generally thought to be lower than that of free cyanide (HCN, CN^−^). Under specific conditions, such complexes can be dissociated, resulting in HCN release, thereby potentially increasing ecosystem contamination (Johnson 2015). Recently, a novel Electro–Fenton system was developed, in which the free cyanides released from iron cyanide (Fe(CN)_6_^3−^) complexes using UV radiation can be effectively converted to nitrate (NO_3_^−^) in the presence of free oxygen radicals (Fig. 2) (Tian et al. 2022). The authors propose that CN^−^ is likely transferred into ^⋅^CN by exposure to hydroxyl radicals (^⋅^OH). Then, the ^⋅^CN intermediates react with superoxide anion radical (O_2_^⋅−^) to form NO_3_^−^, preventing the formation of cyanate anions (CNO^−^) and promoting further CN^−^ uptake or utilisation (Tian et al. 2022).

### Hydrogen cyanide in living organisms

HCN has been described to be synthesised within diverse organisms in all kingdoms except *Archaea* (Díaz-Rueda et al. 2023). In bacteria and fungi, the amino acid glycine is oxidised and decarboxylated by the membrane-bound flavoenzyme cyanide synthase, producing HCN and carbon dioxide (CO_2_) (Knowles 1976; Blumer and Haas 2000). HCN and HCN-containing molecules, including cyanogenic glycosides, also serve as a nitrogen source for the generation of amino acids and other N-containing molecules, but other roles for HCN have been identified or suggested (Díaz-Rueda et al. 2023). The fluorescent pseudomonads emit HCN and have been shown to have important effects against plant diseases, which may be associated with direct poisoning. It has also been shown that these bacteria stimulate plant growth depending on their capacity to emit HCN (Kuzmanović et al. 2018; Sehrawat et al. 2022). Another example is cyanogenic glycosides, which are accumulated in some plants; HCN released from them serves as an immediate chemical defence against herbivores or pathogens that cause damage to the plant tissue (Møller 2010). In case of fungal attack, e.g. on the cyanogenic rubber tree (*Hevea brasiliensis* Muell. Arg.), HCN is liberated from the tissue infected by the *Microcyclus ulei*. HCN disrupts the interactions between the plant host and the fungal pathogen, inhibiting active defence responses that rely on biosynthetic processes once a certain threshold concentration is exceeded (Lieberei et al. 1989). The decomposition of cyanogenic plants or the activity of cyanogenic microbiota (*Chromobacterium*, *Anacystis*, *Nostoc*, *Plectonema*, or free-living forms of *Rhizobium* and rhizosphere *Pseudomonas*) are the source of HCN in the soil (Ebbs et al. 2010).

HCN concentration and duration (*in planta*) depend on the activity of enzymes related to synthesis (described below) and detoxification (*β*-cyanoalanine synthase and rhodanese), as discussed in detail in reviews by Díaz-Rueda et al. (2023) or Krasuska et al. (2023).

#### HCN formation in plants

In non-classical cyanogenic plants, e.g. spinach (*Spinacia oleracea* L.), barley (*Hordeum vulgare* L.), and maize (*Zea mays* L.), HCN concentration was evaluated during synthesis in a variety of systems, including the glyoxylate pathway in the presence of hydroxylamine and manganese ions (Mn^2+^) (Solomonson 1981; Hucklesby et al. 1982). It was revealed in a study on *A. thaliana* that responses to key plant pathogens are linked to the formation of cyanides from tryptophan-derived metabolites, including the camalexin (indolic phytoalexin) biosynthetic pathway (Böttcher et al. 2009). Another pathway of cyanide formation, related to plant defence, involves 4-hydroxyindole-3-carbonyl nitrile (4-OH-ICN) formation, a branch of indole metabolism. Both camalexin and 4-OH-ICN synthesis are dependent on cytochrome P450 activity induced by pathogen attack. The hydrolysis of indole-3-carbonyl nitriles in aqueous solutions also leads to HCN release (Rajniak et al. 2015). The camalexin properties were found to be both antibacterial and antifungal. This molecule disrupts the cell membranes, leading to a reduction of proline uptake and cell viability (Rogers et al. 1996). Camalexin accumulates at high levels near the site of pathogen infection, often surpassing the concentration needed to inhibit pathogen growth in vitro (Rogers et al. 1996; Kliebenstein et al. 2005; Glawischnig 2007). Camalexin can also be subjected to degradation, detoxification and export by pathogens, to attenuate its toxic effects (Pedras and Khan 2000; Pedras and Ahiahonu 2005; Stefanato et al. 2009).

##### HCN and the ethylene biosynthetic pathway interactions

In plants, HCN is produced principally during the biosynthesis of ethylene (ET), which involves the conversion of *S*-adenosyl-l-methionine (SAM) to 1-aminocyclopropane-1-carboxylic acid (ACC, ET direct precursor) by ACC synthase. ACC is then enzymatically converted to ET, HCN and CO_2_ by ACC oxidase (Krasuska et al. 2023). HCN is liberated from the carbon C-1 of the ACC with a stoichiometric ratio of HCN and ET formation of 1:1 (Peiser et al. 1984). ET generation also occurs under non-enzymatic conditions, as observed in an in vitro study by Gniazdowska et al. (2010a), ACC in water solution can be oxidised to ET in the presence of H_2_O_2_, nitric oxide (NO) or HCN. HCN also interacts with the ET signalling pathway, in particular through the regulation of *Ethylene Response Factor1* (*ERF1)* transcription, but does not stimulate the ET production, as demonstrated in sunflower embryos (Oracz et al. 2008). In addition, Smith and Arteca (2000) demonstrated that the ACC synthase gene *ACS6* was activated by HCN in *Arabidopsis thaliana*. The study of Seo et al. (2011) proved that in transgenic rice (*Oryza sativa* ‘Nipponbare’) resistant to blast fungus (*Magnaporthe oryzae*), this resistance was weakened when ET biosynthetic genes were silenced, leading to reduced ethylene production. This compromised resistance was restored by applying KCN externally, but not by ethephon (an ethylene-releasing compound). In a susceptible rice cultivar, treatment with KCN or ACC induced resistance to blast fungus in a dose-dependent manner, whereas ethephon showed no effect (Seo et al. 2011). KCN inhibited blast fungus growth both in vitro and *in planta*. These findings suggest that KCN, produced during the hypersensitive response in infected tissues, plays a key role in rice resistance by limiting fungal growth (Seo et al. 2011). HCN also triggers protein oxidation during sunflower and apple seed dormancy alleviation (Oracz et al. 2007; Krasuska et al. 2014), which may be regarded as one of the mechanisms for seed dormancy alleviation. The mode of action and formation of ethylene oxide (EO), an ET metabolite, is another point for a need to evaluate in plant systems. EO is an industrial gas commonly used to sterilise medical equipment, cosmetics, pharmaceuticals, etc. (Golden and Williams 2014). It is highly toxic to humans (exposure to even a small amount can be fatal) and has been implicated in DNA damage, reduction in reproduction potential and spontaneous abortion, skin irritations and sensitisation, neurological disorders and various types of cancers (Shore et al. 1993; Valdez-Flores et al. 2010; Vincent et al. 2019). Although present in relatively low quantities within plants*,* EO probably plays an important role in plant tissue as a metabolic product in the ET pathway (Jerie and Hall 1978; Dodds et al. 1979; Smith et al. 1985). EO takes part in the modification of protein residues, as was demonstrated for plasma albumin, in which EO reacted with arginyl, cystyl, histidyl, lysyl, methionyl, and tyrosyl residues (Starbuck and Bush 1963). After the reaction of EO with commercial proteins, a decrease in their enzymatic activity was also reported. The reduction of activity was related to electrophilic hydroxyethylation of an atom with one or more lone pairs of electrons, particularly nitrogen and sulphur (Windmueller et al. 1959). A question arises: is there any possibility for HCN interaction with EO or even participation of HCN in the EO formation? It can be postulated that HCN in the presence of ROS may stimulate ET oxidation to EO, but this requires further experimental verification. That may be a significant area of further interest.

##### HCN formation in plants during cyanogenesis

The presence of cyanogenic compounds in the members of the *Fabaceae*, *Poaceae*, *Rosaceae*, and *Asteraceae* families, as well as some conifers and ferns, has been well characterised (Gleadow et al. 2012; Gleadow and Møller 2014; Yulvianti and Zidorn 2021; Myrans et al. 2021; Cowan et al. 2021; Sohail et al. 2023). The *Sapindaceae*, the *Hippocastanaceae*, and the *Boraginaceae* families are also known to store cyanolipids consisting of esterified fatty acids to a mono- or a di-hydroxy nitrile moiety (Avato and Tava 2022). The mechanisms of cyanogenesis are well known and described in detail (Gleadow and Woodrow 2002; Gleadow et al. 2008; Morant et al. 2008; Díaz-Rueda et al. 2023). The cyanogenic compounds constitute an important barrier against animal, microbial, and fungal attack. Cyanogenic glycosides are stored as inactive metabolites that later permit the generation of a variety of bioactive metabolites in herbivores and even other plants, from diverse tissues over time. The activating enzymes causing lysis are very elegantly compartmentalised separately to their substrates so no contact occurs until the plant tissues have been degraded or damaged (Miller and Conn 1980; Gleadow and Møller 2014). Cyanogenic glycosides are enzymatically metabolised via *β*-glucosidases that break the *β*-glycosidic bond. The released *α*-hydroxynitrile is then further converted to HCN and corresponding aldehydes or ketones (Boter and Diaz 2023). Cyanohydrins (hydroxynitriles) are catabolised by hydroxynitrile lyases (HNL) into HCN (Poulton 1990; Zagrobelny et al. 2008). HNL is also involved in the reaction of condensation of HCN with an aldehyde or a ketone, which is the pathway of the cyanohydrins formation (Zagrobelny et al. 2008; Arnaiz et al. 2022).

##### HCN formation in plants from amino acids

Another potential source of HCN in plants is associated with amino acids like histidine, phenylalanine, tyrosine, cysteine and tryptophan which, in the presence of peroxidases, COX, heme- or ferri-cyanide, can be a HCN precursors (Gewitz et al. 1976a, b, 1980; Pistorius et al. 1977, 1979). HCN formation was observed after the addition of various amino acids to *Chlorella vulgaris* extracts and isolated grana from leaves of New Zealand spinach (Gewitz et al. 1976c). In this study, histidine served as a main source of HCN in both *Chlorella vulgaris* extracts and spinach grana. d-histidine was about ten times more effective than l-histidine and histamine, whereas these two isomers (and histamine) were about equally effective with leaf grana. Addition of Mn^2+^, peroxidase, l-tyrosine and l-cysteine also resulted in HCN release from grana, but these amino acids caused little or no HCN formation in *Chlorella* extracts (Gewitz et al. 1976c). Pistorius et al. (1977) in extracts of *Chlorella* observed that the formation of HCN, especially from histidine, but also from other aromatic amino acids, was catalysed by both d-amino acid-oxidase and l-amino acid-oxidase when supplemented with peroxidase or a suitable metal ion such as Mn^2+^.

#### HCN formation in animals

Mammalian cells have been documented to produce HCN from glycine in a reaction catalysed by peroxidases (Stelmaszyńska 1986; Borowitz et al. 1997; Gunasekar et al. 2000). Vinnakota et al. (2012) have shown trace levels of plasma HCN, and it has also been observed that white blood cells produce HCN during phagocytosis (Stelmaszyńska 1986). The role of gut microbiota has not been well characterised in HCN production, although gut microbiota are involved in detoxification of cyanide compounds e.g. in bamboo-eating pandas (Zhu et al. 2018). More recently Zuhra et al. (2025) proposed that glycine may be an endogenous source of cyanide formation in the human and mouse primary hepatocytes and the human hepatoma cell line (HepG2). After glycine supplementation, the highest level of HCN was detected for the lysosomal fraction compared to the cytosol fraction and was linked to high lysosome integrity (Zuhra et al. 2025). The formation of cyanide from glycine requires the oxidation of the amino group to a nitrile group that occurs in the presence of peroxidases, which generates strong oxidants like hypochlorite (HOCl). In the reaction catalysed by myeloperoxidase or peroxidasin, glycine in the presence of H_2_O_2_, Cl^−^ at an acidic pH can be converted to cyanide (in vitro studies). The incubation of glycine with HOCl at pH 4.5, without any enzyme, also led to cyanide generation (Zuhra et al. 2025). As glycine is the common amino acid in plants, this pathway of HCN biosynthesis in the presence of peroxidase class III (POx) in autotrophic cells is feasible and needs further investigation.

### Reactive oxygen species: formation and scavenging

In this review, we are focused on the HCN and ROS cross-talk. The incomplete reduction or excitation of oxygen molecules results in various ROS (radical and non-radical forms) generation (Considine and Foyer 2021). The best-known are O_2_^⋅**−**^, ^⋅^OH and H_2_O_2_, but also peroxyl (ROO^⋅^), alkoxyl (RO^⋅^) and hydroperoxyl (HO_2_^⋅^) radicals (Mittler 2017). Free radicals are generally stable for a very short period (Teixeira et al. 2013) but are the most reactive and therefore dangerous molecules, especially ^⋅^OH. In contrast, H_2_O_2_ is less reactive but more mobile and thus is involved in the transduction of the signal inside and between the cells (Mittler 2017; Van Aken 2021; Mittler et al. 2022).

In excess, ROS are toxic molecules that lead to accelerated ageing and even cell death (Santos et al. 2018; Ciacka et al. 2020; Maldonado et al. 2023). Using the example of seeds, the concept of the “oxidative window”, which emphasises the concentration-dependent physiological function of ROS was created (Bailly et al. 2008) and recently developed (Bailly 2023). This model has the potential for universal application to many physiological processes in plants including stress responses. ROS are implicated in plant growth and development, creating redox potential hubs, cross-talk with hormones, and participating in organelle-to-organelle and cell-to-cell signalling (Mittler 2017; Mittler et al. 2022). As strong oxidants, they influence the structure of molecules and are responsible for post-translational modifications of proteins, leading to both reversible and non-reversible alterations (Ciacka et al. 2020; Martí-Guillén et al. 2022). Redox regulation systems, together with specific proteins and small compounds, are crucial in plants' responses to various signals (e.g. stressors) (Peláez-Vico et al. 2022).

**Fig. 3:**
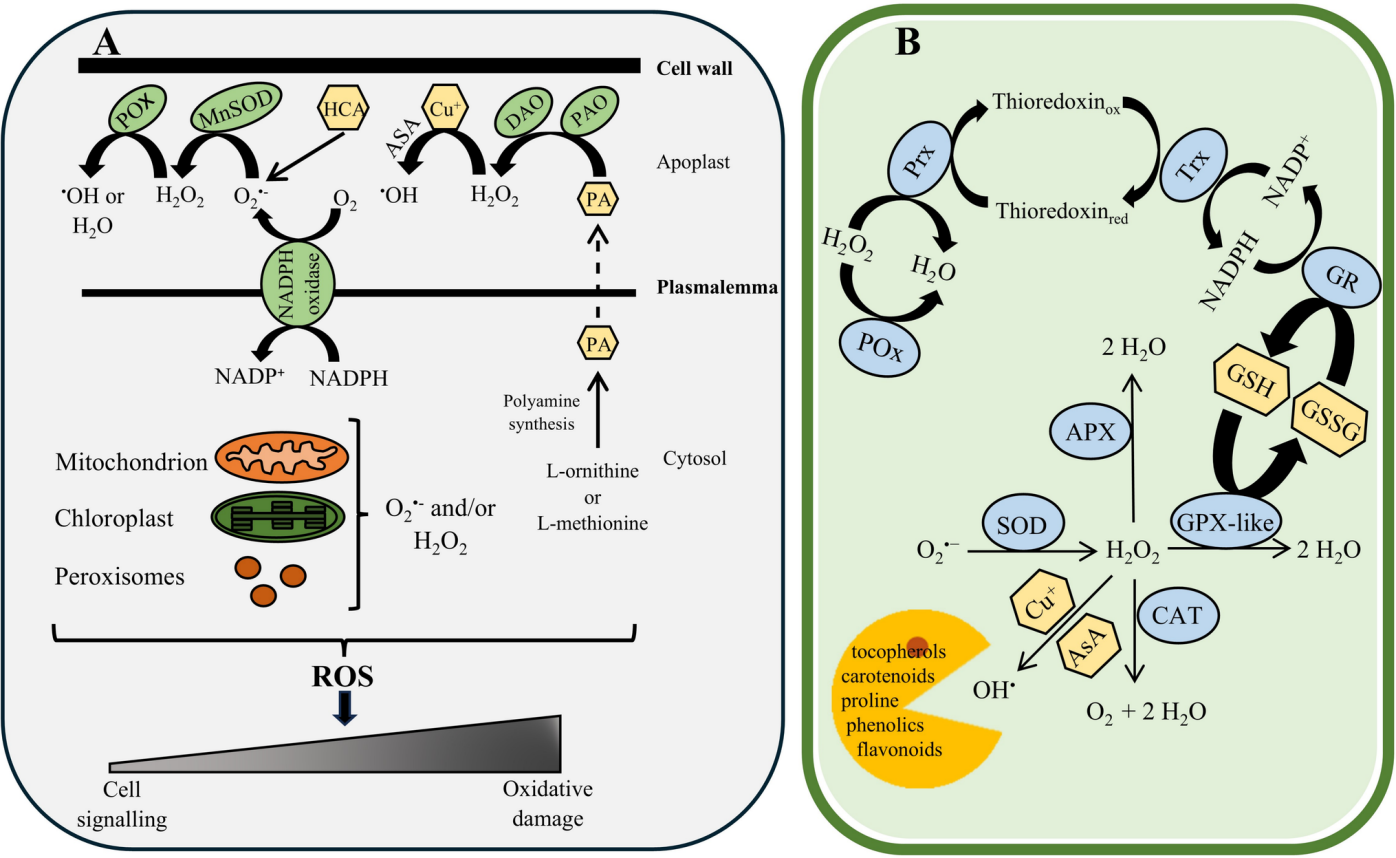
ROS production pathway (**A**) and scavenging system (**B**) in a plant cell. ROS are produced in mitochondria, chloroplasts and peroxisomes, and in the apoplastic space. Cell-wall-bound POx together with MnSOD and transmembrane NADPH oxidase create ^⋅^OH, which also is a product of the reaction of H_2_O_2_ with cell wall-bound Cu^+^ through a Fenton reaction in the presence of ascorbate (ASA). Autoxidation of hydroxycinnamic acid (HCA) results in additional O_2_^⋅−^ generation. One of the H_2_O_2_ synthesis mechanisms involves cell wall-bound DAO and PAO that catabolise polyamines (PA). At the low, controlled level, ROS acts as a signalling molecules regulating cellular processes. ROS over-accumulation due to an inefficient antioxidant system leads to oxidative damages, e.g. lipid peroxidation, DNA oxidation, and protein carbonylation (based on Del Río 2015). The antioxidant system is composed of enzymatic antioxidants and non-enzymatic particles. To the enzymatic antioxidants, superoxide dismutase’s (SOD), peroxidases (POX), catalase (CAT), glutathione peroxidase-like (GPX-like), glutathione reductase (GR), thioredoxins (Trx) and peroxiredoxins (Prx) are implicated. SOD maintains the level of O_2_^⋅−^ in the cell and generates H_2_O_2_, which is then sustained by CAT, APX, and GPX-like enzymes. GPX-like in this reaction also transferred the reduced form of glutathione (GSH) to the oxidised form GSSH. GSSH is then reduced by GR, which involves the reduction of NADPH to NADP^+^. The NADPH pool is restored by Trx, which involves the oxidation–reduction reaction of thioredoxins. Prx is responsible for H_2_O_2_-scavenging resulting in thioredoxin oxidation. POx, same as Prx, is involved in H_2_O_2_ removal. The best known non-enzymatic antioxidants are proline, carotenoids, tocopherols, phenolic compounds and flavonoids, serving as radical suppressors

Plants generate ROS in plastids (mostly chloroplasts), mitochondria, peroxisomes and in the apoplastic space (Palma and Corpas 2021) (Fig. 3A). In plants, chloroplasts in leaves are the main source of ROS under light conditions. In other organisms and non-photosynthesising plant organs, mitochondria play a crucial role in ROS formation. The damage or dysfunction of this cellular organelle is linked to electron leakage increasing O_2_^⋅**−**^ generation. O_2_^⋅**−**^ is converted to H_2_O_2_ by the activity of manganese isoform of superoxide dismutase (MnSOD) (Fig. 3A). The toxicity of O_2_^⋅**−**^ is linked to the damage associated with metallo-sulphur complexes, including the Rieske iron-sulphur protein in Complex III (Van Aken 2021). The formation of ROS in mitochondria was demonstrated for Complexes I and III (Fisher-Wellman and Neufer 2012). Catabolism of polyamines (PA) by amine oxidases: copper-containing diamine oxidase (DAO) and flavin-containing polyamine oxidase (PAO) is an additional source of ROS, particularly H_2_O_2_ (for review, see Pottosin et al. 2014) (Fig. 3A).

The ^⋅^OH production occurs in the presence of (i) ascorbate and Cu as catalysts or (ii) POx (Fig. 3A). Ascorbate non-enzymatically reduces O_2_ to H_2_O_2_ and Cu^2+^ to Cu^+^; the reaction of H_2_O_2_ with Cu^+^ results in ^⋅^OH generation through a Fenton reaction (Fry 1998; Schopfer 2001). High concentrations of Cu in the cell wall can decrease the contribution of cell wall peroxidases in ^⋅^OH production (Hadži-Tašković Šukalović et al. 2010). POx participate in both H_2_O_2_ detoxification and ^⋅^OH production (Hadži-Tašković Šukalović et al. 2010; Miura 2012). In a recent study using pea (*Pisum sativum* L.) cell wall fragments isolated from roots of 14-day-old plants, POx was shown to cooperate with cell wall-bound SOD to generate ^⋅^OH. The autoxidation of hydroxycinnamic acid in the cell wall leads to O_2_^⋅−^ production (Fig. 3A). Subsequently, O_2_^⋅−^ is converted to H_2_O_2_ by cell wall-bound MnSOD. H_2_O_2_ could then be converted to ^⋅^OH by POx (Kukavica et al. 2009).

Maintaining the ROS level for effective signalling requires efficient systems (both enzymatic and non-enzymatic) for producing and scavenging these molecules. The proteins responsible for control of ROS levels include various isoforms of SOD, catalase (CAT), ascorbate peroxidase (APX), glutathione peroxidase-like (GPX-like), glutathione reductase (GR), thioredoxins, glutaredoxins, and peroxiredoxins (Fig. 3B) (Ciacka et al. 2020; Klupczyńska et al. 2022; Peláez-Vico et al. 2022). POx action is typically bimodal. These heme-containing enzymes detoxify H_2_O_2_ to water using phenolic compounds (Takahama and Oniki 2000). POx’s may use thiol or salicylic acid as substrates, and free radicals are generated as a result of the derivative radical’s reaction with O_2_ (Shigeto and Tsutsumi 2016). Non-enzymatic compounds that participate in the modulation of ROS content include a reduced form of ascorbic acid and a reduced form of glutathione (GSH), plus proline, carotenoids, and *α*-tocopherols (Fig. 3B) (Hernández et al. 2009; Signorelli et al. 2014; Morscher et al. 2015; Noctor et al. 2024). Besides glutathione and ascorbate, phenolic compounds and flavonoids assist in maintaining cell redox homeostasis (Fig. 3B) (Hernández et al. 2009; Eilenberg et al. 2010). Modulators and ROS sensors such as GPX-like, peroxiredoxin, cysteine-rich receptor like kinase 5 (CRK), and leucine-rich-repeat receptor-like kinase (HPCA1), are the basis of effective plant defence, adaptation, and tolerance (Fichman et al. 2022; Hilleary 2022; Peláez-Vico et al. 2022). CRKs are known to play important roles in plant immunity and defence responses to abiotic stressors and in the control of plant development (Krasensky-Wrzaczek and Wrzaczek 2024).

### HCN and ROS cross-talk in plant and animal cells

Despite differences between plant and animal systems, similarities have been observed in the cells of all organisms concerning the mutual relations of cyanide and ROS. At the cellular level, cyanide not only alters the activity of enzymatic antioxidants, especially those with Fe or Cu in the active centre, but also impacts the content of cellular antioxidants such as GSH, proline or melatonin (see below).

#### HCN–ROS cross-talk in mitochondrial respiration

The inhibition of COX activity by HCN binding is accompanied by the rapid accumulation of ROS. This is especially true for higher (above 100 µM) concentrations of HCN in animal cells. In contrast, the application of KCN from nanomolar to low micromolar concentrations to mammalian cells stimulated mitochondrial respiration, promoted cell proliferation, and even protected cells via the modulation of ROS level (Correia et al. 2012; Randi et al. 2021). In plants, the effect of HCN on the electron transport chain in mitochondria is altered by the presence of alternative enzymes. The inhibition of COX induces the activity of the HCN-insensitive alternative oxidase (AOX) (Bogatek et al. 1991; El-Khoury et al. 2022). The alternative pathway involving AOX allows electron flow from reducing equivalents to O_2_ generating a “safety values” to prevent excess ROS (Van Aken 2021; Li et al. 2024). As previously demonstrated for embryos of dormant apple (*Malus domestica* Borkh.) seeds, short-term pre-treatment with 1 mM HCN resulted in only slight inhibition of respiration (at the beginning of the culture in the light), but 4 days later, stimulation of oxygen uptake took place (Bogatek et al. 1991). The pre-treatment of tomato (*Solanum lycopersicum* L.) seeds with 10 μM KCN had a stimulatory effect on total respiration rate and AOX pathway respiration (Yu et al. 2022). In tomato plants, inoculation by *Tobacco mosaic virus* (TMV) induced systemic defence characterised as increased accumulation of AOX gene transcript levels (*AOX1a-c*) and enhanced NO generation. Spraying leaves with KCN at nonlethal concentrations (1 mM) or NO donor diethylamine NONOate (DEA/NO) also induced an accumulation of *AOX1* transcript, and reduced TMV viral RNA accumulation (Fu et al. 2010). The discovery of cyanide-insensitive cellular respiration in a thermogenic plant revealed a potential connection between the alternative respiration pathway and the regulation of floral thermogenesis (Watling et al. 2008; Miller et al. 2011; Roemer et al. 2013; Ito-Inaba et al. 2019). AOX mediates this alternative pathway, which diverges from the cytochrome pathway at the ubiquinone pool. In this process, ATP production is reduced due to decreased proton pumping along the electron transport chain, and the energy difference between ubiquinol and O_2_ is released as heat (Watling et al. 2008). In the study by Li et al. (2022) on lotus (*Nelumbo nucifera* Gaertn.), the authors observed that mitochondria in thermogenic cells were more dynamic, abundant, and morphologically diverse compared to those in non-thermogenic cells. Moreover, the increased activity of mitochondria led to elevated ROS production, which triggered a significant upregulation of AOX in the respiration pathway. This, in turn, helped remove excess ROS, maintaining homeostasis in thermogenic cells during floral thermogenesis (Li et al. 2022). There is a gap in our knowledge linking the metabolism of thermogenic plants to cyanide action. Furthermore, the stimulatory effect of cyanide at nanomolar concentration on mitochondrial Complex IV was noted in mammalian cells treated with diluted supernatant of *Pseudomonas aeruginosa* (a cyanide-producing bacterium) (Randi et al. 2021). The opposite effect was achieved when concentrated supernatants were used. The authors proposed that the cyanide-stimulating effect was related to the removal of the constitutive, inhibitory glutathionylation on COX catalytic 30- and 57-kDa subunits.

#### HCN action is linked to the alterations in ROS level in plants

HCN mode of action is dependent on ROS levels and the concentration of antioxidants and their activity. The pre-conditioning of cucumber (*Cucumis sativus* L. cv. Jihong no. 2) seedlings with KCN (20 µM) solution by spraying the leaves, 24 h before stress treatment: salt (200 mM NaCl), PEG (16% PEG6000) or cold (4 °C) strengthened the plant resistance to all stressors (Xu et al. 2012). In contrast, KCN at higher concentrations (from 0.05 to 1.0 mM) had no positive effect, even resulting in oxidative stress generation, observed as an increase in H_2_O_2_ content (Xu et al. 2012). Pre-treatment of cucumber seedlings with 20 µM KCN solution before stress application was beneficial to plants and stimulated the AOX pathway and ET biosynthesis. The authors suggest that KCN at low concentrations did not lead to ROS overaccumulation. ROS content modulation via HCN may be due to the activation of antioxidants. Indeed, under stress conditions, in HCN pre-treated cucumber seedlings, the activities of GPX-like and APX were significantly higher (Table 1). A higher ratio of reduced to oxidised glutathione (GSH/GSSG) and ascorbate/dehydroascorbate was also noted (Xu et al. 2012). The alterations in the GSH/GSSG ratio are not surprising and are likely due to the reactivity of HCN (the cleavage of disulphide bonds in proteins and peptides) (Catsimpoolas and Wood 1966).

**Table 1:** Summary information on the effect of cyanide on the activity of individual enzymes of ROS metabolism in plants

Enzyme	Effect	Cellular localisation	References
CAT	**Stimulation** after 24 h of pre-treatment of apple embryos (6 h, 1 mM KCN)	Peroxisomes	Oracz et al. (2009), Krasuska and Gniazdowska (2012), Gerivani et al. (2019)
	**Inhibition** in protein extract (isolated from sunflower embryos) after treatment with 5–100 µM KCN (in vitro studies)		
	**No effect** just after pre-treatment of apple embryos (6 h, 1 mM KCN) and walnut kernels (4 h, 1 mM KCN)		
POx	**Stimulation** after 24 h of pre-treatment of apple embryos (6 h, 1 mM KCN); after 6 days of pre-treated walnut kernels (4 h, 1 mM KCN)	All compartments	Krasuska and Gniazdowska (2012), Gerivani et al. (2019)
	**Inhibition** just after pre-treatment of apple embryos (6 h, 1 mM KCN) and walnut kernels (4 h, 1 mM KCN)		
APX	**Stimulation** after pre-treatment of cucumber seedlings before stress induction (spraying with 20 µM KCN); after 6 days of HCN pre-treated walnut kernels (4 h, 1 mM KCN)	All compartments	Xu et al. (2012), Gerivani et al. (2019)
	**No effect** in walnut embryos just after 4 h pre-treatment with 1 mM KCN		
GPX-like	**Stimulation** just after and after 24 h of pre-treatment of apple embryos (6 h, 1 mM KCN): after pre-treatment of cucumber seedlings before stress induction (spraying with 20 µM KCN)	Cytosol	Xu et al. 2012; Krasuska and Gniazdowska 2012
GR	**Stimulation** after 24 h of pre-treatment of apple embryos (6 h, 1 mM KCN)	All compartments	Oracz et al. (2009), Krasuska and Gniazdowska (2012)
	**Inhibition** just after pre-treatment of apple embryos (6 h, 1 mM KCN)		
	**No effect** in protein extract (isolated from sunflower embryos) after treatment with KCN at the range 0–1 mM (in vitro studies)		
SOD	**Stimulation** after 24 h of pre-treatment of apple embryos (6 h, 1 mM KCN)	FeSOD (chloroplast)	Oracz et al. (2009), Krasuska and Gniazdowska (2012), Gerivani et al. (2019)
	**Inhibition** walnut kernels just after pre-treatment with 1 mM KCN, 4 h; in protein extract (isolated from sunflower embryos) after treatment with KCN at the range 0.1–1 mM (in vitro studies)	Cu/ZnSOD (chloroplast, cytosol)	
	**No effect** just after pre-treatment of apple embryos (6 h, 1 mM KCN)	MnSOD (mitochondrion, peroxisomes)	
NADPH oxidase	**Stimulation** after 6 days after pre-treatment of walnut kernels (4 h, 1 mM KCN)	Transmembrane protein	Gerivani et al. (2019)
	**No effect** in walnut embryos just after pre-treatment with (4 h, 1 mM KCN)		

KCN exposition was at room temperature for apple embryos, at 10 °C for sunflower and at 27 °C for walnut kernels

Cyanide may influence redox potential by lowering the GSSG concentration. This relationship has been observed during seed dormancy removal (Oracz et al. 2008, 2009; Gniazdowska et al. 2010a; Krasuska and Gniazdowska 2012). HCN treatment of sunflower embryos overcomes dormancy by increasing the content of H_2_O_2_ and O_2_^⋅**−**^ in the embryonic axes (Oracz et al. 2009). Short-term (6 h) pre-treatment of dormant apple embryos (which accumulate cyanogenic compounds) with HCN stimulated germination and undisturbed development of the seedlings (Bogatek et al. 1991). This beneficial effect was (among other mechanisms) linked to ROS metabolism. The concentration of H_2_O_2_ and O_2_^⋅−^ in apple embryos and embryonic axes transiently increased due to fumigation with 1 mM HCN (Gniazdowska et al. 2010a; Krasuska et al. 2014). Moreover, HCN pre-treatment increased H_2_O_2_ secretion into the imbibition medium of apple embryos (Gniazdowska et al. 2010b). The lack of symptoms of oxidative stress in apple embryos implies that the action of HCN is associated with the induction of H_2_O_2_ signal, both short-distance (within the cell) and long-distance (cell–cell, organ-organ, or organ-environment). The germination of dormant Persian walnut (*Juglans regia* L.) kernels shortly fumigated with 1 mM HCN was accompanied by the increase of O_2_^⋅−^ in the radical part of the embryonic axis, and transient H_2_O_2_ content just after the treatment (Gerivani et al. 2019). Similarly, the addition of cyanide at low concentrations in dormant cardoon (*Cynara cardunculus* L. var. sylvestris) promoted ROS accumulation in embryonic axes (Puglia et al. 2022). The positive effect of tomato seeds pre-treatment with 10 μM KCN on germination rate was linked to the increase of H_2_O_2_ after 24 to 48 h of the culture (Yu et al. 2022). Moreover, after 60 h of tomato seed germination, a notable decrease in H_2_O_2_ content was also observed (Yu et al. 2022). Thus, cyanide potentially acts beneficially as a pre-treatment agent in plants exposed to various abiotic stressors and stimulates seed germination and seedling development via modulation of cellular ROS level.

#### Examples of HCN–ROS interaction in animal cells

The breakthrough discovery of Zuhra et al. (2025) shows that low concentrations of cyanide are generated endogenously in various mammalian tissues and cells. The authors detected cyanide in several cellular compartments of human cells and in various tissues and the blood of mice. At a specific rate, cyanide exerts stimulatory effects on mitochondrial bioenergetics, cell metabolism and cell proliferation, but at high concentrations, it impairs cellular bioenergetics.

The cytoprotective action of KCN treatment was demonstrated for rat brain endothelial (RBE4) and NT2 neuron-like cells against high glucose-induced damage, leading to apoptotic cell death (Correia et al. 2012). The authors linked the cyanide mode of action to the ROS level in the functional mitochondria and the hypoxia-inducible factor-1 alpha (HIF-1α) signalling pathway. Low levels of cyanide (below 1 μM) and a short term of exposure (1 h) were sufficient to increase ROS content, mostly H_2_O_2_, and at the same time, an increase in oxygen consumption was detected. Moreover, the free radical scavenger N-acetyl cysteine and the specific HIF-1α inhibitor 2-methoxyestradiol totally blocked the positive effect of low-dose cyanide treatment (Correia et al. 2012). Extended exposure of Syrian hamster embryo (SHE) cells to a higher concentration of HCN had a carcinogenic effect (Kamendulis et al. 2002). HCN at 0.5 mM concentration resulted in the morphological transformations of SHE cells, accompanied by DNA oxidation, noted as an increase in 8-hydroxy-2ʹ-deoxyguanosine (a mutagenic metabolite). Moreover, the induction of morphological transformation was reduced by vitamin E in a dose-dependent manner (Kamendulis et al. 2002). Treatment of Rhesus monkey kidney epithelial cells (LLC-MK2) with KCN at poisoning dose (0.31–20 mM) for 2–24 h was accompanied by ROS and reactive nitrogen species (RNS) over-generation (Hariharakrishnan et al. 2009). The authors observed lower mitochondrial activity, lower ATP concentration, together with higher ROS/RNS production, and oligonucleosomal DNA fragmentation and nuclear fragmentation. Furthermore, lower GSH content and lower activity of SOD and CAT were noted, especially when KCN at higher concentrations was used. Such cellular disturbances were prevented using *α*-ketoglutarate (metabolite used as an antidote in cyanide poisoning) and N-acetyl cysteine (Hariharakrishnan et al. 2009). Thus, exposure of animal cells to low doses of cyanide stimulates ROS production and has a positive effect. On the other hand, treatment with cyanide of higher doses caused irreversible changes that reduced cell viability, resulting in potential death. HCN meets many of the general criteria of a ‘classical’ mammalian gasotransmitter and shares several characteristics with established members of this group, such as NO, carbon monoxide (CO), and hydrogen sulphide (H_2_S) (Zuhra and Szabo 2022).

### HCN action is linked to the ROS modulators’ activity in plants and animal systems

#### HCN influence antioxidant enzymes activity

ROS level depends on their production and scavenging by the cellular antioxidant system. The maintenance of ROS balance after the HCN pre-conditioning of apple embryos resulted from the stimulation of antioxidant enzyme activities (Krasuska and Gniazdowska 2012). Just after the treatment (6 h), decreased POx and GR activities were noted, while the activity of total SOD and CAT were similar compared to the control. In contrast, HCN stimulated GPX-like activity (Table 1). Furthermore, 24 h after the pre-treatment, all analysed enzymatic activities were higher than those of the control (Krasuska and Gniazdowska 2012). The decrease in POx activity after HCN pre-treatment may be due to direct impacts of HCN on enzyme activity. As it belongs to the class III peroxidases, it is a heme-containing glycoprotein that lacks strict specificity against reductants (Hiraga et al. 2001). Besides cyanides, ^⋅^CN is also able to inhibit POx (Chen et al. 2000). The SOD isoforms are linked to the specific metal ions present at the active site: copper/zinc (Cu/ZnSOD), manganese MnSOD, and iron (FeSOD) (Bowler et al. 1994). The best-known isoform of SOD, which is HCN and H_2_O_2_ sensitive, is Cu/Zn SOD, while MnSOD is resistant to both inhibitors. FeSOD (from chloroplast) is sensitive only to H_2_O_2_ (Bowler et al. 1994). In sunflower axes of embryos treated with HCN, CAT and SOD activities were inhibited (while the GR activity was not affected) (Table 1) (Oracz et al. 2009). Moreover, the stimulatory effect of HCN was potentially linked to the nicotinamide-adenine dinucleotide phosphate (NADPH) oxidase—a membrane-bound enzyme complex involved in the O_2_^⋅**−**^ generation. The germination of dormant sunflower embryos pre-treated with HCN in the presence of diphenyleneiodonium (DPI, an inhibitor of NADPH oxidase) has been reduced by 40% (Oracz et al. 2009). Free radicals, especially ^⋅^OH, participate in the loosening of the cell wall (Fig. 3A). Thus, HCN via ROS (free radicals) may be involved in the root protrusion during seed germination (Liszkay et al. 2004) or in the cell wall thickening (generally via H_2_O_2_) to prevent pathogen attack (Kärkönen and Koutaniemi 2010). HCN pre-treatment of walnut kernels initially decreased the activity of NADPH oxidase (Gerivani et al. 2019). At the same time, lower activity of SOD and POx were detected with no effect on the activity of CAT and APX. After 4 days of the treatment, enhanced activities of POx, APX and NADPH oxidase in embryonic axes were noted (Table 1).

#### Mode of action of cyanide in regulation of activity of enzymatic antioxidants

**Fig. 4:**
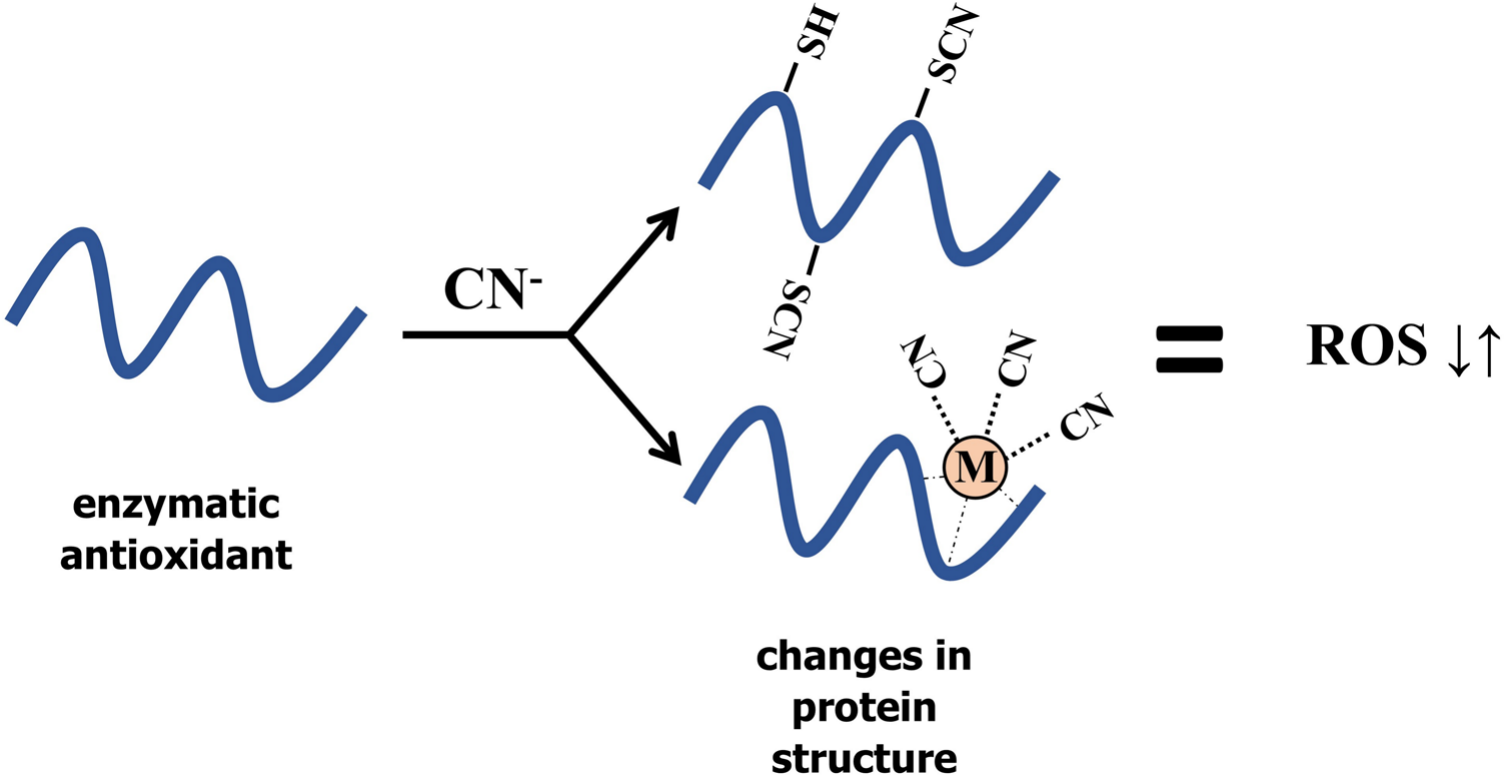
HCN regulates ROS metabolism by alterations in the activities of enzymatic antioxidants, which in turn provoke ROS level perturbations (lowering (↓) or increasing (↑) ROS content). Changes in enzymatic activity due to cyanide action are related to protein post-translational modification by *S*-cyanylation of cysteine residues (-SCN) and/or possible complexation with transition metal ions (M) of the prosthetic groups of metalloproteins

Cyanide can react with transition metals (including Cu) to form cyanide metal complexes with coordination numbers from two to eight (Hanusa 2020). When cyanide is present in the cellular environment, it can form a very stable complex with transition metals of the prosthetic groups of metalloproteins that are crucial for protein activity (Nagahara et al. 1999) (Fig. 4). The treatment with 50 mM KCN of ^75^seleno-labelled GPX isolated from ovine erythrocytes resulted in the activity loss and release of selenium (Prohaska et al. 1977). This inhibitory effect was dependent on KCN dose, temperature-, pH-, time-, and oxidation-state of the enzyme. The oxidation of GPX enables the cyanide to inhibit the enzyme. As mentioned, HCN fumigation of dormant apple embryos stimulated GPX-like activity (Table 1) (Krasuska and Gniazdowska 2012). As plants do not have classical GPX, but the GPX-like (a protein without selenocysteine), this enzyme may be less sensitive to this gasotransmitter.

Variable activity of some enzymes of the antioxidant system in the presence of HCN was also observed in animal tissues. KCN (0.5 mM) lowered CAT activity in SHE cells but only after 4 h and 1 day of the treatment. When KCN application took place for 2 or 7 days, KCN stimulated CAT activity (Kamendulis et al. 2002). Similarly, SOD activity decreased after 4 h and 1 day of KCN (0.5 mM) treatment; however, after 2 and 7 days, it increased to the value of the control (Kamendulis et al. 2002). The diverse effects of cyanide on CAT activity in plant’s material (Table 1) are most probably linked to the H_2_O_2_ concentration in the cells. A ferric protoporphyrin IX (namely heme* b*) is located in the active centre of CAT. The nitrogen of HCN blocks heme access to other potential ligands. HCN interacts with the distal histidine and asparagine residues, thus competing with H_2_O_2_ for heme binding (Glorieux and Calderon 2017). However, the cyanide inhibition is apparently non-competitive when H_2_O_2_ concentrations reach lower values. Only at high concentrations of H_2_O_2_ the inhibition becomes competitive (Ogura and Yamazaki 1983). Thus, HCN stimulates SOD, POx, CAT, and GPX-like activities differentially due to diversity in the structure of the active centre and/or the presence of an appropriate metal.

The interactions of NaCN with Cu-containing amine oxidase (CuAO) from pea seedlings and *Arthrobacter globiformis* have been investigated (Shepard et al. 2004). CuAOs are homodimers (70 to 95 kDa per subunit) and each contains a single active site composed of a Cu ion and the 2,4,5-trihydroxyphenylalanine quinone (TPQ) as cofactor (Dove and Klinman 2001; Dawkes and Phillips 2001). The mechanism of CuAO inhibition by NaCN was proposed to occur through complexation to Cu(I), thereby directly competing with O_2_ for reoxidation of TPQ. Despite cyanohydrin derivatisation of the TPQ cofactor in these enzymes, the non-competitive inhibition of amine oxidation is determined to arise almost exclusively through the cyanide complexation of Cu(I) (Shepard et al. 2004).

As HCN affects enzymatic antioxidants, feedback loops may modify the enzyme's activities (directly or by post-translational modifications) (Fig. 4). In Arabidopsis APX (APX1), NADPH-depended thioredoxin reductase (NTR2) and POx were subjected to* S*-cyanylation of cysteine residues—one of the protein post-translational modification generated by cyanide (García et al. 2019) (Fig. 4). We propose that the HCN–ROS cross-talk depends on the cellular redox potential, which functions as a powerful signal per se and the current status of reactive metabolites in plant cells under certain physiological conditions.

### Neurotransmitters and proline in HCN–ROS action

**Fig. 5:**
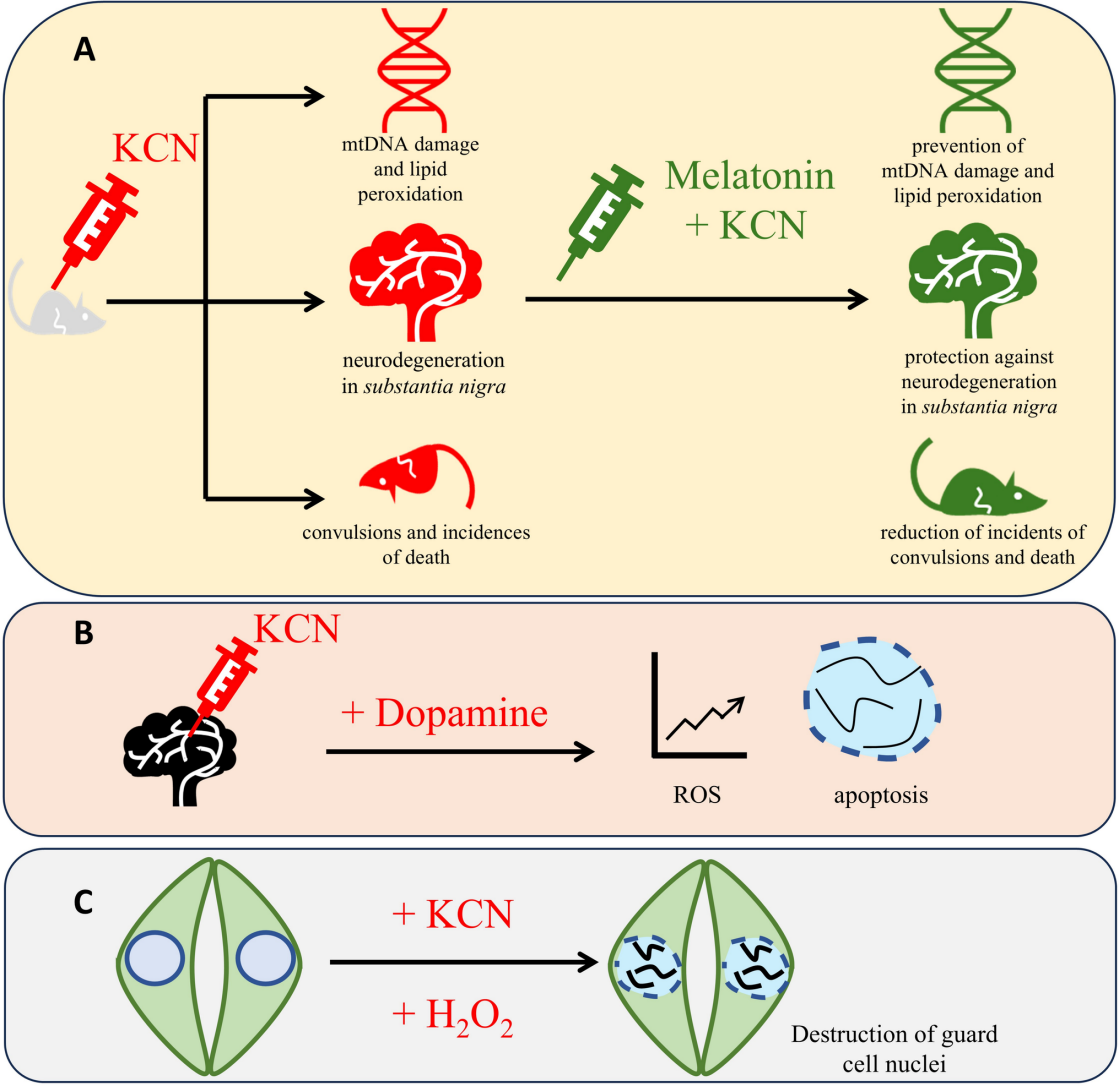
The positive effect of melatonin on KCN-induced mtDNA damage, lipid peroxidation, neurodegeneration of *substantia nigra*, convulsions and incidences of death in ICR mice (**A**), negative effect of cyanide by enhancement of dopamine-induced ROS accumulation and apoptosis in mesencephalon cells (**B**) and harmful effect of simultaneous application of KCN and H_2_O_2_ leading to destruction of guard cell nuclei in pea leaves (**C**)

N-acetyl-5-methoxytryptamine, also known as melatonin, is not only an animal neurotransmitter but also functions as a plant growth and development regulator (Arnao and Hernández-Ruiz 2015; Khan et al. 2024; Kolupaev et al. 2024). This molecule directly scavenges ROS, both radical and non-radical forms. Furthermore, melatonin maintains ROS levels by regulating of activity of antioxidant enzymes such as SOD, POx, CAT, APX, GR or other cellular antioxidants such as ascorbate, GSH, flavonoids, and anthocyanins (for references, see Gu et al. 2022). It is known that melatonin action is also related to other gasotransmitters, like NO, CO or H_2_S (Kolupaev et al. 2024). However, to date, there are a lack of data referring to the role of melatonin in plant’s response to cyanide. However, in animal-based research, such reports are found. In male Institute of Cancer Research (ICR) mice (widely used, outbred strain of mice in biomedical research), melatonin (at a dose of 20 g per kg) significantly reduced the incidence of convulsion induced by KCN injection (at a dose of 4, 5, 6, and 7 mg per kg). Melatonin also inhibited cyanide-induced acute lethality and protected against neurodegeneration of *substantia nigra* cells (Fig. 5A) (Choi and Han 2002). In another study, the effect of KCN on mitochondrial DNA (mtDNA) in mouse brain was investigated in vivo and in vitro (Yamamoto and Mohanan 2002). In vitro experiments indicated that incubation of crude mitochondrial fraction from mouse brain with KCN (0.1, 1 and 2 mM) led to the damage of mtDNA and increased lipid peroxidation (0.1 and 1 mM KCN), which were not observed after co-treatment with melatonin (1 and 1.5 mM) (Fig. 5A). After adding H_2_O_2_ (1.5 mM) and Fe^2+^ (^⋅^OH generating mixture) to brain mtDNA, the same effect was noticed, which was diminished by co-treatment with melatonin. In mice, subcutaneous injection of KCN (7 mg per kg) caused both brain mtDNA damage and severe seizures, which were prevented after melatonin (20 mg per kg) pre-injection (in vivo studies) (Fig. 5A) (Yamamoto and Mohanan 2002). These data suggest that ROS, mainly ^⋅^OH, are involved in mtDNA damage induced by KCN in animals, and melatonin is a mandatory molecule that protects against this damage.

HCN toxicity in animals may also be accelerated by other signalling compounds, strengthening its ability for oxidative stress induction, as demonstrated by dopamine-induced apoptosis in primary cultured mesencephalon cells (Jones et al. 2003). The authors demonstrated that the application of 300 mM dopamine for 24 h stimulated apoptosis due to ROS formation. The tissue pre-treatment with 100 mM KCN for 30 min before dopamine application enhanced this apoptotic effect by ROS overaccumulation (Fig. 5B). However, KCN at the same concentration alone did not induce apoptosis (Jones et al. 2003). Samuilov et al. (2000) have used KCN as a programmed cell death (PCD) inducer in plants, and observations under light microscopy indicated that cyanide application caused fragmentation and destruction of nuclei in cells of epidermis isolated from pea leaves. In darkness or light, H_2_O_2_ enhances the cyanide-induced destruction of guard cell (GC) nuclei (Fig. 5C). Significant stimulation of this process was observed after addition of 10 μM H_2_O_2_, and almost 100% destruction of nuclei was achieved at 100 μM H_2_O_2_. Hydrogen peroxide did not induce nucleus destruction without KCN, even at 10–50 mM concentrations (Samuilov et al. 2006). This data suggests the engagement of both KCN and ROS in nuclei decomposition in leaves.

Catecholamines, like dopamine or epinephrine, are also synthesised in plant cells (Liu et al. 2020a). The concentration and synthesis of dopamine in plants vary depending on the species and growth conditions. Various abiotic stressors are known to induce dopamine synthesis, which, in turn, decreases ROS content via the stimulation of antioxidant activity (Liu et al. 2020a). Despite great interest in catecholamines in plants, relatively little is known about these modulators. It is also unknown whether HCN could stimulate dopamine synthesis or whether this catecholamine implicates HCN biosynthesis. The linkage of catecholamines to HCN–ROS cross-talk in plants requires further investigation.

Proline action as a beneficial agent in stress-exposed plant tissue relates to its ROS scavenging properties and increased antioxidant capacity of the cells (Hayat et al. 2012; Mohammadrezakhani et al. 2019). Leaf senescence relates to both ET and ROS formation, thus in senescence tissue beside ET also HCN evolution occurs. Senescence of rice leaves in darkness resulted in elevated proline level, whereas KCN application at high, toxic concentrations (1 mM or 100 mM) decreased proline content (Wang et al. 1982). Alternations in proline content after cyanide exposure were also described in rice seedlings in the context of nutrition with NO_3_^**−**^ or ammonium (NH_4_^+^). Increased ammonium feeding led to enlarged proline level in the shoot, with no effect in the root. When CN^**−**^ was used as an additional N source (during ammonium feeding), the proline level decreased to some extent (Liu et al. 2020b). When KCN solutions (0.44 mg L^−1^, 0.93 mg L^−1^, 1.44 mg L^−1^ or 1.95 mg L^−1^) were added into the ammonium-free medium (with NO_3_^**−**^ as a main N source), the increased accumulation of proline in roots of rice seedlings was detected. In shoot tissues KCN only at the highest (1.44 mg L^−1^ and 1.95 mg L^−1^) doses induced proline accumulation (Yu et al. 2014). As KCN at all tested concentrations lowered the relative growth rate of the seedlings (Yu et al. 2014), it may be proposed that proline accumulation may reduce the negative impact of cyanide toxicity.

## Conclusions and perspectives

Although HCN has been considered a toxic substance for many years, recent studies suggest its regulatory nature in both plants and animals. Besides directly regulating the cell’s energy status, HCN acts via secondary messengers belonging to ROS (e.g. H_2_O_2_). Cyanide influences the redox potential of the tissue. HCN's ability to inhibit/stimulate enzymatic activity depends on the oxidation state of the target protein and its metal ion in prosthetic group. Sensitivity to HCN and then cellular response is also dependent on the developmental stage of the organism and its current physiological conditions, particularly exposure to significant environmental stressors, inducing secondary oxidative stress. In this way, cyanide action is similar to other well-known gasotransmitters, e.g., NO.

The effect of cyanide on plants has not been fully investigated, and reviewing the literature reveals many gaps that present opportunities for future research. The potential role of cyanide in regulating autophagy and apoptosis, such as in the endosperm of germinating seeds, appears to be an emerging area of research. Describing the potential impact of cyanide in the activation of polyamine catabolic and anabolic pathways may point to the not-yet-described polyamine-cyanide crosstalk of great importance in plant development. As mentioned before, NaCN inhibits the activity of CuAO, which indicates that cyanide may impact the polyamine catabolic pathway and may impair NO generation, a crucial molecule in plant physiology that cooperates with ROS and impacts cellular antioxidant system. Moreover, the roles of melatonin, dopamine, and proline in response to cyanide application warrants further investigation as these compounds are important but still less known plant regulators.

## Data Availability

This study did not involve the use of additional data.
